# Improvement in gravel-mulched land soil nutrient and bacterial community diversity with *Lonicera japonica*

**DOI:** 10.3389/fmicb.2023.1225503

**Published:** 2023-12-07

**Authors:** Xing Wang, Bin Ma, Hua Liu, Yangmei Bao, Ming Li, Neil B. McLaughlin, Lanping Guo

**Affiliations:** ^1^Institute of Forestry and Grassland Ecology, Ningxia Academy of Agricultural and Forestry Sciences, Yinchuan, China; ^2^Chengdu Institute of Biology, Chinese Academy of Sciences, Chengdu, China; ^3^State Key Laboratory for Quality Ensurance and Sustainable Use of Dao-di Herbs, National Resource Center for Chinese Materia Medica, China Academy of Chinese Medical Sciences, Beijing, China; ^4^Ottawa Research and Development Centre, Agriculture and Agri-Food Canada, Ottawa, ON, Canada

**Keywords:** gravel-mulched land, *Lonicera japonica*, soil physical and chemical indicators, enzyme activity, bacterial community

## Abstract

Gravel-mulched land in China suffers from poor natural resources and fragile ecological environment, posing a challenge to effective restoration of ecological function. *Lonicera japonica,* a traditional Chinese herb used for treating human diseases, is a highly adaptable and resilient plant species, can effectively improve the soil properties, and may have important implications for the ecology and economy of gravel-mulched land. A study was conducted in a gravel-mulched field to measure the impact of planting the *L. japonica* (including control (CK), 1-year, 2-year, and 4-year cultivation of plants) on (i) dynamic changes in soil nutrient and enzyme activity properties, and (ii) soil rhizosphere microbial community structure characteristics. We found that the concentration of soil organic carbon, available nitrogen, available phosphorus and available potassium in *L. japonica* soil after cultivation for 1–4 years increased by 11–409%. The urease, phosphatase and catalase activities were increased by 11–560%, with the highest nutrient concentration and enzyme activity in 4-year plants. The pH value gradually decreased after cultivation. The improved soil environments increased soil bacterial community diversity. Planting *L. japonica* significantly increased the bacterial ACE, Chao1 index, Simpson index, and Shannon-Wiener index. The *Firmicutes*, *Proteobacteria* and *Bacteroidetes* were observed in dominant phyla. The relative abundance of eight genera, including *Streptococcus*, *Veillonella* and *Rothia*, was significantly reduced by more than 1%. Taken together, these soil indicators suggest that planting *L. japonica* in the short term would be a cost-effective strategy to combat soil degradation in a gravel-mulched ecosystem.

## 1 Introduction

It is well-established that soil nutrients, microorganisms and enzymes are important components of soil ecosystems (Na et al., 2021; Li Y. et al., 2023). In soil ecosystems, the rhizosphere is closely related to plants and interacts with them throughout their life cycle (Liu et al., 2019). An increasing body of evidence suggests that the diversity of rhizosphere soil microorganisms and the presence of specific genera can ensure proper soil function and promote plant health (Chen et al., 2019; Zhang et al., 2019). Soil enzymes act as biocatalysts, and their activity level reflects soil biological activity and fertility (Gregorich et al., 1994; Mi et al., 2018). Changes in soil microbial and enzyme activities are important indicators of the effectiveness of soil nutrient fertility utilization and respond rapidly to changes in soil environmental conditions, providing an early indication of changes in soil health (Li J. et al., 2023).

The arid and semi-arid areas of north-west China have a poor natural resource endowment and a fragile ecological environment, and are subject to serious soil erosion, accounting for their poor economic status. The past few years have witnessed a burgeoning interest in restoring the natural habitat of this region. Gravel mulching which began in the early Kangxi period of the Qing Dynasty (1,644 to 1911) dating back to about 300 years ago, is a unique form of drought-resistant farming in arid and semi-arid areas of northwest China (Zhao et al., 2017). Gravel mulching involves spreading a mixture of sand and gravel washed down from alluvial fans to form a 10 to 20 cm thick surface layer on farmland (Zhao et al., 2016, 2020). This method can effectively inhibit evaporation, and improve water storage and moisture conservation. Gravel-mulched land in China is mainly distributed in the arid and semi-arid climate transition zone of Gansu and Ningxia provinces. The vegetation types are mostly desert or desert steppe in this region. Vegetation degradation and land desertification have seriously affected the socio-economic development of the region. Since 2004, the Chinese government has widely promoted this technology by means of subsidies and encouraged planting watermelon (*Citrullus lanatus* (Thunb.) Matsum. & Nakai) on gravel-mulched fields. Because of the long sunshine hours and the large temperature difference between day and night during the growth period, the watermelon grown from the stone crevices is crisp., fleshy and high in sugar (Ren et al., 2023). At the same time, it absorbs the trace element selenium in the sand and gravel, and is actively sought after in the market, which in turn has given birth to a unique selenium sand melon industry. However, long-term watermelon continuous cropping leads to drastic shifts in soil bacterial community composition (Gu et al., 2022), soil mineral deficiencies, and the ecological environment was severely negatively impacted. Watermelon production has declined (Wu et al., 2022), and people began to look for other crops to grow.

*L. japonica* commonly known as “Ren Dong,” has been widely used in traditional medicine in East Asian countries for thousands of years. Its dry flower buds, leaves, and stems are rich in flavonoids, polysaccharides and other components, which have a broad spectrum of antioxidant, anti-carcinogenic, anti-inflammatory, and anti- bacterial effects (Yang et al., 2017; Tang et al., 2021). *L. japonica* extract is widely used in cosmetics, pharmacological preparations, food, and animal husbandry (Li et al., 2020; Zhang X. et al., 2022; Yang et al., 2023). *L. japonica* has strong adaptability (Thanabhorn et al., 2006; Yan et al., 2016), is tolerant of severe cold and drought, and does not require high soil nutrient conditions. Cultivation of *L. japonica* can effectively improve soil nutrients, prevent soil erosion, and fertilize the soil, and has great potential for improvement of the ecological environment and economic development of the gravel-mulched land (Thanabhorn et al., 2006; Yang et al., 2017). Because of its good economic benefits and the emergence of obstacles associated with continuous watermelon production, local enterprises began planting 33.3 ha of *L. japonica* in 2017, and the sales amounted to more than 960,000 yuan in 2018, reaching a gratifying achievement of one-year investment recovery; in 2019, the planting area was expanded to 66.7 ha. The local government began to encourage the people living locally to plant *L. japonica*. By 2023, the cropped area of *L. japonica* was 2000 ha, with a yield of more than 600 kg•ha^−1^ of high-quality dried flowers, a total output value of nearly 192 million yuan. *L. japonica* has truly become the ‘rich flower’ of the masses in Zhongwei City, Ningxia, China.

Currently, research on gravel-mulched land has primarily centered on the effects of soil water infiltration and evaporation (Xie et al., 2006), soil nutrients (Qiu et al., 2015), fertilization (Bao et al., 2022) and sand mulching (Qiu et al., 2023) on crops. Our hypothesis was that planting *L. japonica* will lead to longer-term positive effect on soil microbes, soil enzyme activity and nutrient cycling. To test this hypothesis, a field experiment was conducted in a typical gravel-mulched land to compare growing *L. japonica* for 1 year, 2 years and 4 years with a control with natural shrub vegetation. The specific objectives of the current study were to determine the effects of different years of growing *L. japonica* on (i) the soil properties, and (ii) the composition of the bacterial community of *L. japonica* rhizosphere soil in a gravel-mulched land.

## 2 Materials and methods

### 2.1 Site description

The research site is in the Xiangshan gravel-sand area (E105°15′, N36°06′) of Zhongwei City, Ningxia, China, which has a classic continental weather pattern with 189.5 mm precipitation and 2,400 mm potential evapotranspiration. In this field, the soil structure is loose, water is easily infiltrated and susceptible to evaporation, leading to droughts, sand storms, soil desertification and other natural disasters. The natural vegetation coverage is generally less than 20%, and the vegetation types are dominated by herbs and dry shrubs with low and highly unstable productivity. Land in the Xiangshan gravel-sand area has been used to for monoculture production of watermelon. Since 2017, local enterprises have gradually switched to planting *L. japonica.* The variety of *L. japonica* in the test site was “Beihua No. 2,” provided by Zhongwei Sunshine Muchang Agriculture and Pasture Company Ltd.

### 2.2 Experimental design and field management

**Figure 1:**
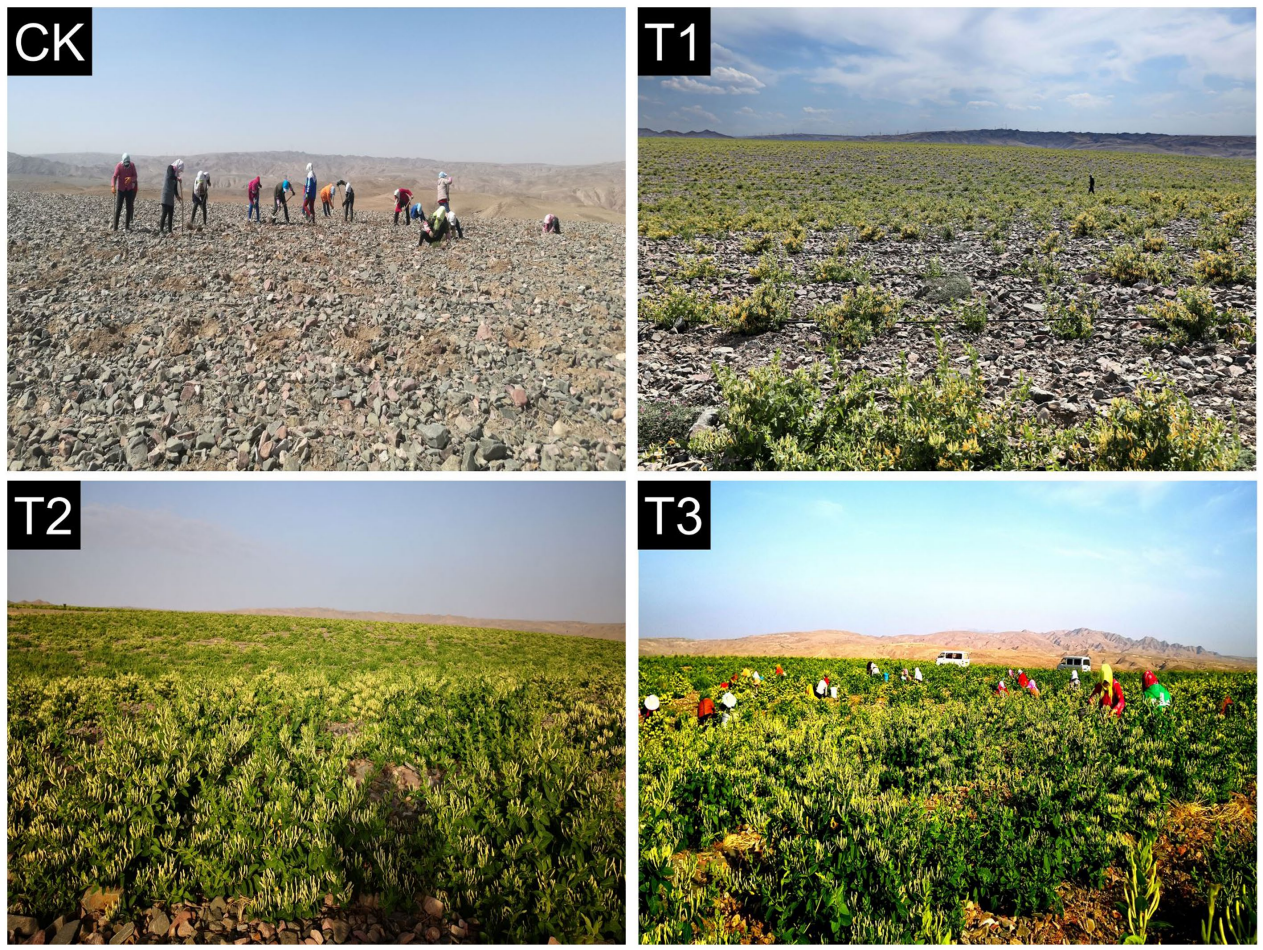
Different years of *L. japonica* cultivation in the experimental field.

The experimental design was a randomized complete block (RCB) design with 3 replicates and four treatments; each plot had dimensions of 5.8 m × 4.4 m. The treatments were control or check with no *L. japonica* cuttings planted (CK), and *L. japonica* cuttings planted in different years: 2020 (T1), 2019 (T2) and 2017 (T3) (Figure 1). Plots were covered by about 20-cm-deep layer of gravel (5–8 cm diameter) mulch. *L. japonica* cuttings (30 cm height) were manually transplanted each year at the end of March to the field after cultivation. Transplanting consisted of digging a hole depth approximately 20 cm, putting the cuttings into hole vertically, backfilling with loose fine soil and compacting, and then covering with a thin layer of gravel-sand. Because of extreme water shortage, limited or no irrigation was applied throughout the growing season, watering was only done 24 h and 1 week after transplanting. Row spacing was 2 m, plant spacing was 1 m, and the planting density was 5,000 plants•ha^−1^. The experiment followed local recommended management practices for weed control and other agronomic operations.

### 2.3 Sample collection

In May 2021, the plant residue and stones were removed from the soil surface and soil samples were collected from 0–20 cm soil layers using a soil auger (5 cm internal diameter) and three sampling points were randomly chosen between two adjacent rows within each plot (Ma et al., 2022), The collected soil samples were passed through a 2 mm mesh sieve and divided into two parts. One part was frozen at −80°C to determine the soil microbial microbiome, while the other part was air-dried indoors to determine other soil factors.

### 2.4 Determination of soil characteristics

The soil pH was measured by PHS-3C (Leici, China). The SOC (soil organic carbon) concentration was measured by an Elemental rapid CS cube analyzer (Elementar, Germany). The AN (available nitrogen) concentration in soil was measured by the alkaline diffusion method. The AP (available phosphorous) concentration was measured by NaHCO_3_ leaching and an AA3 flow analyzer (Seal, Germany). Finally, the AK (available potassium) concentration was measured by NaOH fusion-flame photometry (Pansu and Gautheyrou, 2007). Soil phosphatase, catalase and urease activities were measured by disodium phenyl phosphate colorimetry, potassium permanganate titration and indophenol-blue colorimetry, respectively (Guan et al., 1986).

### 2.5 Soil microbial community analysis

The genomic DNA was extracted by the CTAB or SDS method, and then the purity and concentration of DNA were detected by agarose gel electrophoresis. The diluted genomic DNA was used as a template. Primers were 515F and 806R. Phusion ® High-Fidelity PCR Master Mix with GC Buffer and high-efficiency high-fidelity enzyme (New England Biolabs, United States) were used for PCR. PCR products were detected by 2% agarose gel electrophoresis. The target bands were recovered using the gel recovery kit provided by QIAGEN. The library was constructed using the TruSeq ® DNA PCR-Free Sample Preparation Kit. The constructed library was quantified by a Qubit fluorometer and qPCR. Illumina NovaSeq 6,000 platform (Biomarker Technologies, Beijing, China) was used for sequencing.

### 2.6 Data analysis and processing

The data presented in this study were expressed as mean ± standard deviation, and statistical analyses were conducted in R software. Differences in soil properties, enzyme activities and bacterial diversity were calculated with a one-way analysis of variance (ANOVA). The alpha-diversity was evaluated using the ACE, Shannon, Simpson and Chao1 diversity indices. To examine the correlation between soil characteristics and the diversity of the soil microorganism community and the abundance of dominant phyla, Canoco5.0 and “vegan” packages were utilized for redundancy analysis (RDA) and Mantel test. Throughout this paper, probability levels of *p* < 0.05 and < 0.01 were considered to be statistically significant and highly significant, respectively.

## 3 Results

### 3.1 Chemical indicators and enzyme activities

With an increase in cultivation years, there was a significant decrease in soil pH, while the other soil characteristics showed an annual increase (Table 1). The soil pH in CK and T1 (both 9.70) was significantly higher than that in T2 and T3. The SOC in T3 and T2 increased by 70 and 65% compared with CK, respectively. All of AN, AK and AP concentrations were highest in T3, while CK exhibited the lowest levels. Moreover, both urease and phosphatase activities were highest in T3, and were significantly higher by 560 and 37% compared with CK. In contrast, catalase activity was comparable in T3, T2, and T1, and were significantly higher by 31–35% compared with CK.

**Table 1:** Physico-chemical indicators and enzyme activities of soils in different cultivation years.

Treatments	pH	total organic carbon (g•kg^−1^)	Available nitrogen (mg•kg^−1^)	Available phosphorus (mg•kg^−1^)	Available potassium (mg•kg^−1^)	Urease activity [mg•(g•d)^−1^]	Phosphatase activity [mg• (g•d)^−1^]	Catalase activity [mL•(g•20 min)^−1^]
CK	9.70 ± 0.06a	0.70 ± 0.05b	14.87 ± 1.00c	16.02 ± 1.08c	80.29 ± 5.41c	0.05 ± 0.00c	2.74 ± 0.18b	34.78 ± 2.34b
T1	9.70 ± 0.06a	0.92 ± 0.06ab	31.31 ± 2.05b	17.93 ± 1.18c	132.70 ± 8.72b	0.18 ± 0.01b	3.06 ± 0.20ab	45.74 ± 3.00a
T2	9.20 ± 0.06b	1.16 ± 0.08a	31.20 ± 2.04b	31.36 ± 2.06b	137.75 ± 9.04b	0.23 ± 0.02ab	3.47 ± 0.23ab	45.89 ± 3.01a
T3	9.00 ± 0.06c	1.19 ± 0.23a	44.39 ± 3.70a	81.54 ± 4.46a	172.90 ± 9.45a	0.33 ± 0.06a	3.77 ± 0.31a	47.10 ± 0.92a

The means in the same column followed by the same letter indicate no significant differences according to the Tukey test for multiple comparisons (*p* > 0.05).

### 3.2 OTUs analysis of the microbial community

**Figure 2:**
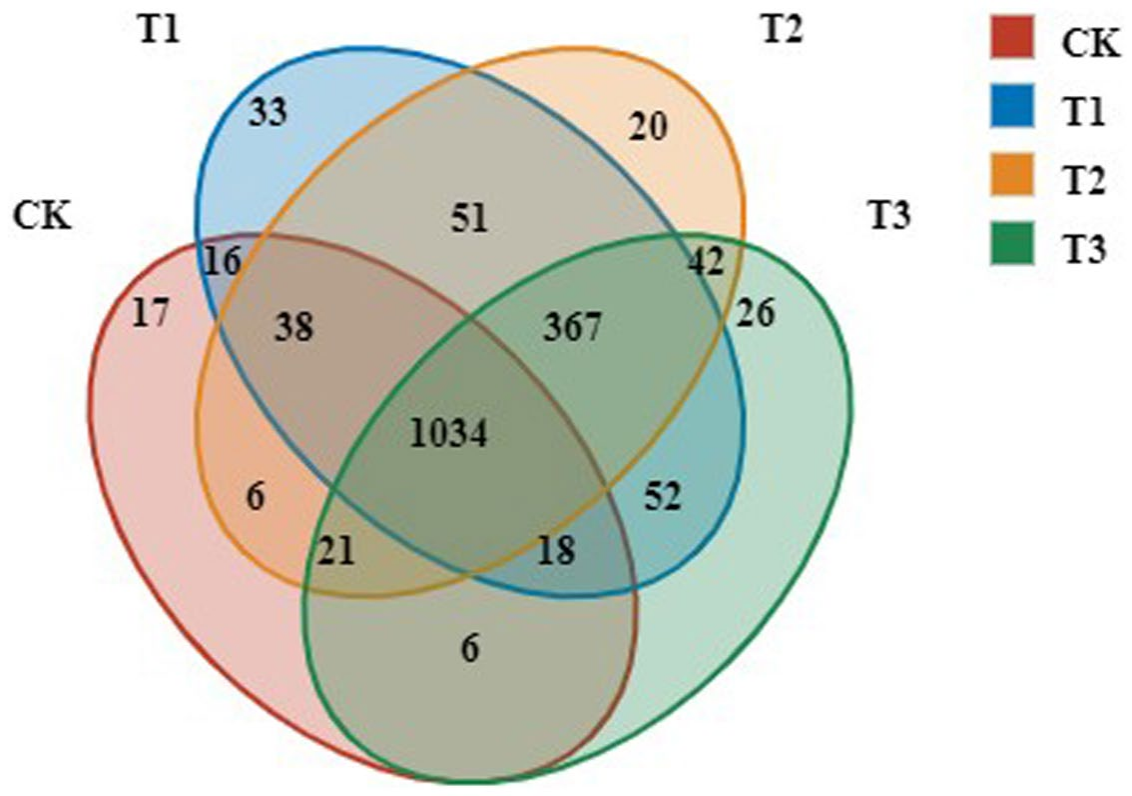
OTUs analysis of bacteria in rhizosphere soil following different years of *L. japonica* cultivation.

To identify the dominant OTUs in different treatments, both the overlap and the distribution of the 5,910 most abundant OTUs across all samples were determined (Figure 2). Venn diagram analysis identified 1,566, 1,579, 1,609 and 1,156 OTUs in the T3, T2, T1 and CK groups, respectively. There were 1,034 common OTUs and 17, 33, 20, and 26 unique OTUs, accounting for 1.66, 1.27, 2.05, and 1.47% of total OTUs in T3, T2, T1 and CK groups, respectively. The number of OTUs in T3, T2 and T1 increased significantly compared with CK.

### 3.3 Composition of the bacterial community

**Figure 3:**
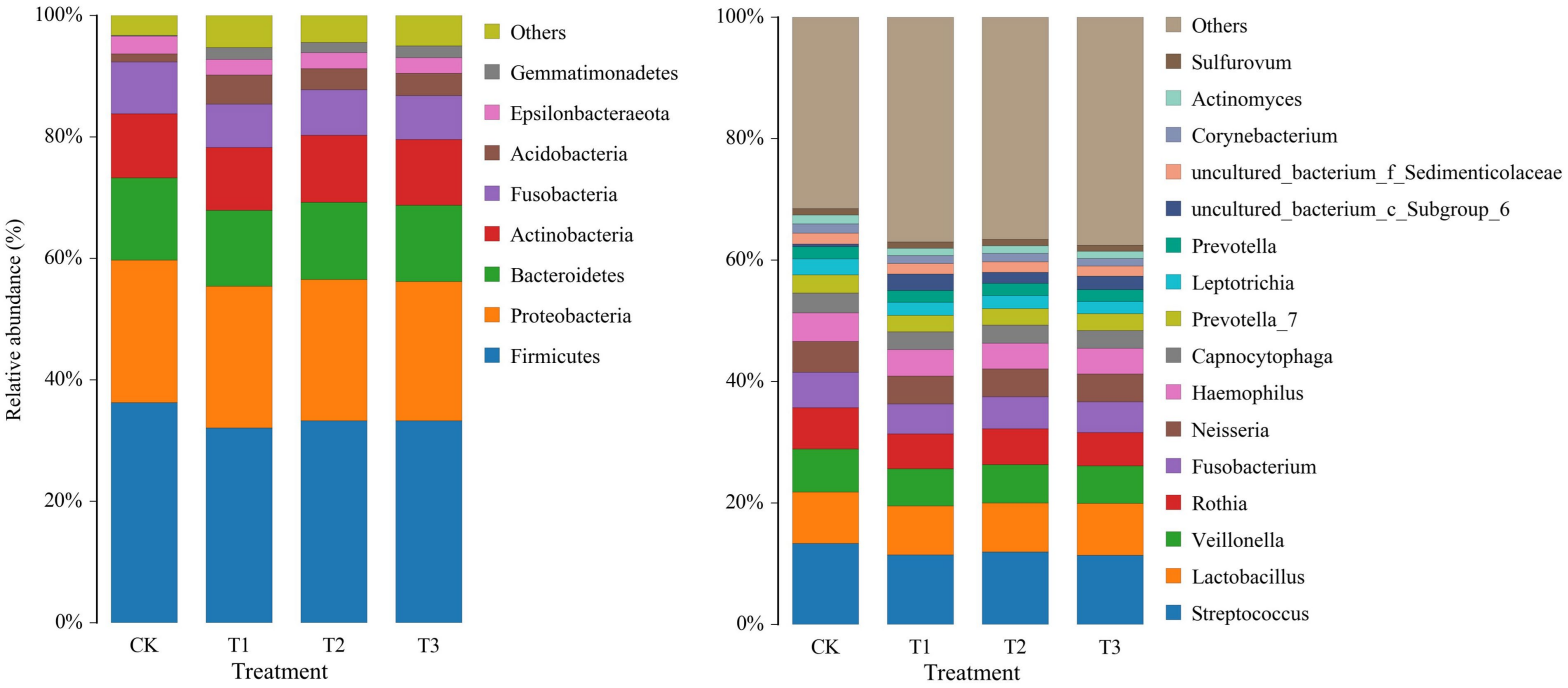
Relative abundance of bacteria at phylum (left) and genus (right) levels in rhizosphere soil following different years of *L. japonica* cultivation.

To study the differences in soil microbial communities following different years of *L. japonica* cultivation, the dominant bacteria with a relative abundance of more than 1% were plotted (Figure 3). The CK, T1, T2 and T3 soil bacteria comprised 8 phyla, including *Firmicutes*, *Proteobacteria*, *Bacteroidetes*, *Actinobacteria*, *Fusobacteria*, *Acidobacteria*, *Epsilonbacteraeota* and *Gemmatimonadetes*, accounting for 94.73–96.72% of the relative abundance. Among them, the relative abundance of *Firmicutes*, *Bacteroidetes* and *Fusobacteria* was highest in CK (36.24, 13.52 and 8.55%) and lowest in T2 (32.07, 12.48 and 7.16%, respectively). The abundance in CK was significantly higher than in T2. The relative abundance of *Acidobacteria* was highest in T2 (4.82%), and was significantly higher than CK (1.30%) (Table 2).

**Table 2:** Relative abundance of bacteria at phylum levels in rhizosphere soil as affected by different years of *L. japonica* cultivation.

Treatment	CK	T1	T2	T3
Firmicutes	36.24 ± 0.22a	32.07 ± 1.28b	33.27 ± 1.19ab	33.25 ± 1.13ab
Proteobacteria	23.49 ± 0.45a	23.34 ± 0.54a	23.26 ± 0.23a	22.9 ± 0.07a
Bacteroidetes	13.52 ± 0.17a	12.48 ± 0.33b	12.72 ± 0.33b	12.6 ± 0.25b
Actinobacteria	10.58 ± 0.20a	10.36 ± 0.16a	11.01 ± 0.31a	10.81 ± 0.14a
Fusobacteria	8.55 ± 0.36a	7.16 ± 0.05b	7.54 ± 0.34b	7.21 ± 0.23b
Acidobacteria	1.3 ± 0.22b	4.82 ± 1.18a	3.43 ± 0.62ab	3.76 ± 0.80ab
Epsilonbacteraeota	2.89 ± 0.10a	2.53 ± 0.14a	2.64 ± 0.05a	2.56 ± 0.20a
Gemmatimonadetes	0.15 ± 0.03b	1.98 ± 0.61a	1.7 ± 0.43a	1.93 ± 0.41a
Chloroflexi	0.56 ± 0.15a	1.11 ± 0.23a	1 ± 0.23a	1.21 ± 0.26a
Patescibacteria	0.91 ± 0.04a	0.93 ± 0.02a	0.88 ± 0.02a	0.89 ± 0.01a
Others	1.82 ± 0.11b	3.23 ± 0.41a	2.56 ± 0.14ab	2.9 ± 0.26a

The means in the same line followed by the same letter indicate no significant differences according to the Tukey test for multiple comparisons (*p* > 0.05).

The relative abundances of the bacterial population in the rhizosphere of *L. japonica* which were greater than 1% at the genus level, were classified and were composed of 16 genera (Figure 3). In comparison with the control group (68.44%), the relative abundance of T1 (62.94%), T2 (63.38%), and T3 (62.42%) decreased by 5.06–6.02%, indicating that the relative abundance of other soil bacterial genera increased in T1, T2 and T3. The relative abundances of *Streptococcus*, *Veillonella*, *Rothia*, *Fusobacterium*, *Neisseria*, *Leptotrichia*, *Corynebacterium* and *Actinomyces* were highest in CK (13.32, 7.11, 6.80, 5.80, 5.04, 2.61, 1.55 and 1.39%, respectively). The relative abundances of *Fusobacterium*, *Corynebacterium* and *Actinomyces* were significantly higher in CK than T1 and T2. The relative abundance of the remaining bacteria was significantly higher in CK than in T1, T2 and T3 (Table 3).

**Table 3:** Relative abundance of bacteria at genus levels in rhizosphere soil as affected by different years of *L. japonica* cultivation.

Treatment	CK	T1	T2	T3
Streptococcus	13.32 ± 0.43a	11.46 ± 0.40b	11.94 ± 0.27b	11.41 ± 0.45b
Lactobacillus	8.47 ± 0.28a	8.05 ± 0.35a	8.05 ± 0.66a	8.55 ± 0.24a
Veillonella	7.12 ± 0.07a	6.08 ± 0.33b	6.31 ± 0.12b	6.16 ± 0.20b
Rothia	6.80 ± 0.18a	5.81 ± 0.32b	5.94 ± 0.10b	5.48 ± 0.21b
Fusobacterium	5.80 ± 0.18a	4.93 ± 0.02b	5.24 ± 0.33ab	5.05 ± 0.16b
Neisseria	5.04 ± 0.07a	4.56 ± 0.13b	4.60 ± 0.12b	4.58 ± 0.19b
Haemophilus	4.76 ± 0.04a	4.38 ± 0.27a	4.20 ± 0.16a	4.23 ± 0.16a
Capnocytophaga	3.26 ± 0.17a	2.88 ± 0.07a	2.98 ± 0.09a	2.94 ± 0.09a
Prevotella_7	2.97 ± 0.11a	2.76 ± 0.05a	2.71 ± 0.09a	2.77 ± 0.05a
Leptotrichia	2.61 ± 0.18a	2.11 ± 0.04b	2.17 ± 0.02b	2.03 ± 0.08b
Prevotella	2.04 ± 0.09a	1.96 ± 0.11a	2.02 ± 0.10a	1.94 ± 0.06a
uncultured_bacterium_c_Subgroup_6	0.38 ± 0.11b	2.75 ± 0.80a	1.80 ± 0.42ab	2.21 ± 0.57ab
uncultured_bacterium_f_Sedimenticolaceae	1.84 ± 0.09a	1.71 ± 0.11a	1.73 ± 0.09a	1.64 ± 0.10a
Corynebacterium	1.56 ± 0.06a	1.32 ± 0.04b	1.41 ± 0.07ab	1.26 ± 0.08b
Actinomyces	1.39 ± 0.02a	1.15 ± 0.08b	1.26 ± 0.01ab	1.16 ± 0.06b
Sulfurovum	1.08 ± 0.05a	1.04 ± 0.09a	1.03 ± 0.02a	1.01 ± 0.06a
Others	31.55 ± 1.28b	37.06 ± 1.53a	36.61 ± 1.63a	37.58 ± 1.50a

The means in the same line followed by the same letter indicate no significant differences according to the Tukey test for multiple comparisons (*p* > 0.05).

### 3.4 Bacterial community diversity

**Figure 4:**
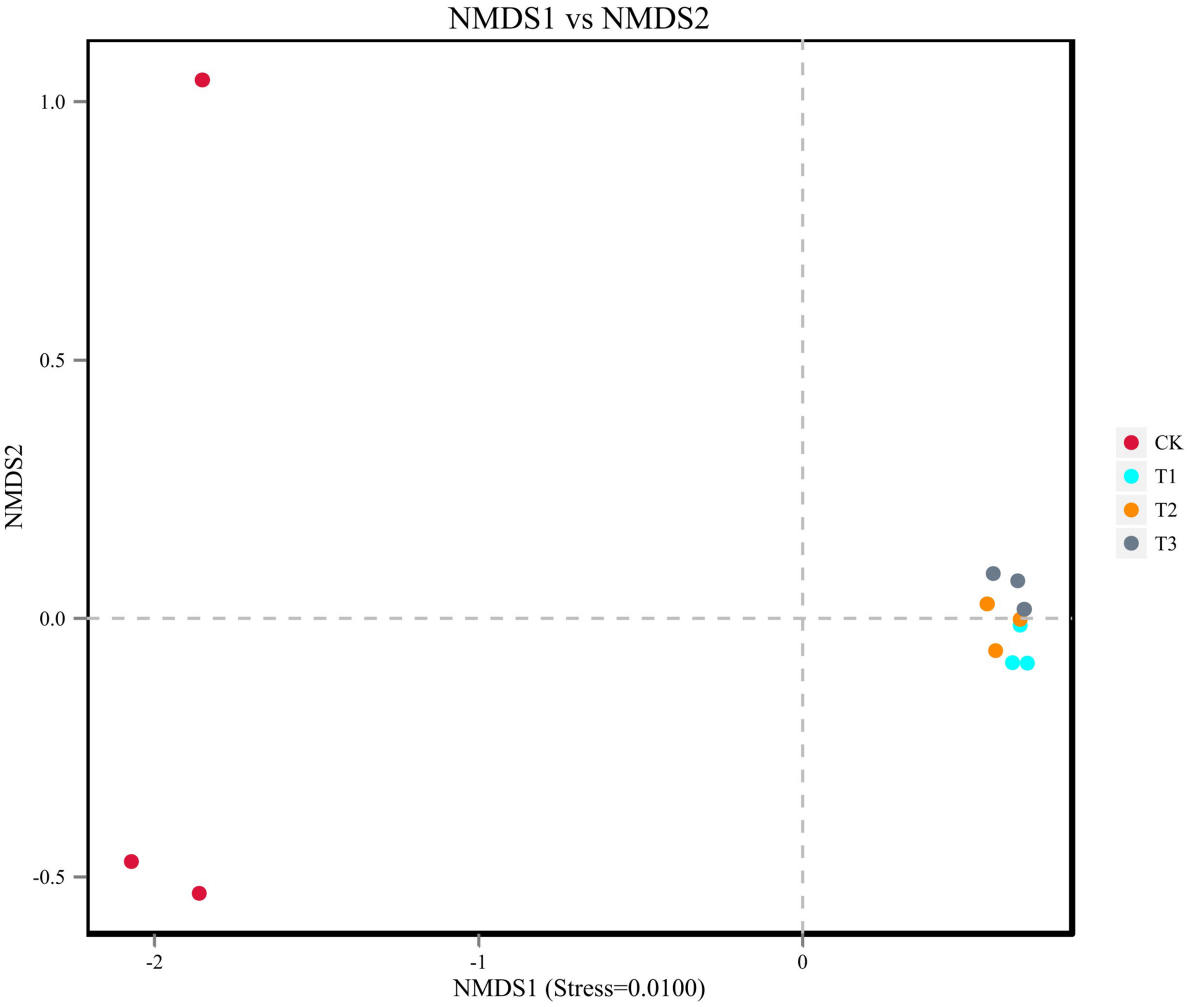
Beta diversity was analyzed using NMDS ordinations of Binary_Jaccard similarities calculated based on relative OTU abundances of the four different treatments.

The soil bacterial diversity indices for different years of cultivation showed the same trend (Table 4). In T1, T2 and T3, the soil bacteria ACE index (1,503, 1,465 and 1,487), the Chao1 index (1,520, 1,495 and 1,505), the Simpson index (all 0.97), and the Shannon-Wiener index (all 7.0) were significantly higher than CK. The planting *L. japonica* formed a cohesive group, which was significantly separated from the treatment with no *L. japonica* cuttings planted (CK) (Figure 4).

**Table 4:** Soil bacterial community diversity following different years of cultivation.

Treatments	ACE index	Chao1 index	Simpson index	Shannon-Wiener index
CK	1048.44 ± 45.90b	1104.90 ± 43.29b	0.96 ± 0.00b	6.56 ± 0.09b
T1	1502.98 ± 24.07a	1520.32 ± 30.62a	0.97 ± 0.00a	7.24 ± 0.16a
T2	1464.73 ± 31.75a	1495.39 ± 38.37a	0.97 ± 0.00a	7.14 ± 0.16a
T3	1487.28 ± 43.78a	1504.90 ± 45.54a	0.97 ± 0.00a	7.23 ± 0.15a

The means in the same column followed by the same letter indicate no significant differences according to the Tukey test for multiple comparisons (*p* > 0.05).

### 3.5 Redundancy analysis of bacteria and soil factors

**Figure 5:**
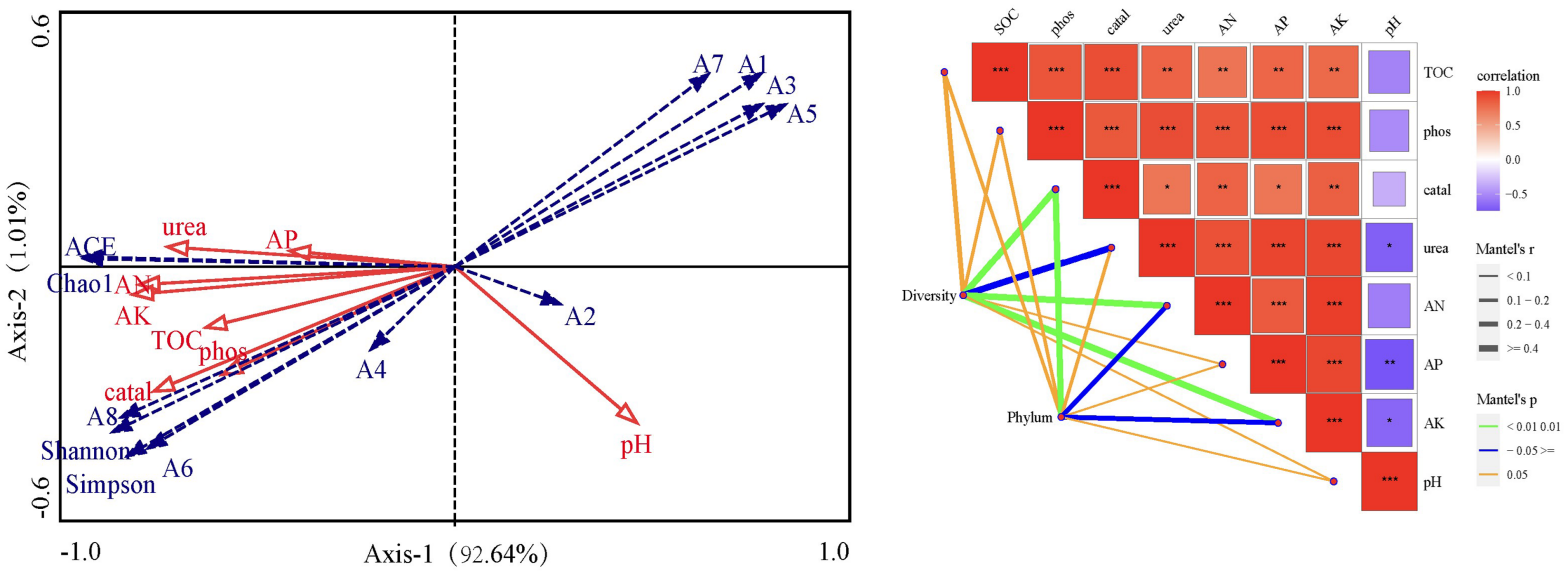
Redundancy analysis and mantel test plot of dominant bacterial community and environmental factors. SOC, Soil organic carbon; AN, Available nitrogen; AP, Available phosphorus; AK, Available potassium; Urea, Urease activity; Phos: Phosphatase activity; Catal, Catalase activity; A1 ~ A8, *Firmicute*, *Proteobacteria*, *Bacteroidetes*, *Actinobacteria*, *Fusobacteria*, *Acidobacteria*, *Epsilonbacteraeota*, *Gemmatimonadetes*; ACE, ACE index; Shannon, Shannon-Wiener index; Simpson, Simpson index; Chao1, Chao1 index.

The redundancy analysis showed significant relationships among soil organic carbon, available nitrogen, available phosphorus, available potassium, urease activity, phosphatase activity, catalase activity, and bacterial community diversity (Figure 5). All soil parameters were positively correlated with each other, except pH which was negatively correlated with the other parameters. The phyla *Actinobacteria*, *Acidobacteria* and *Gemmatimonadetes* were positively correlated with soil catalase, phosphatase and urease activity and SOC, AK and AP, and were negatively correlated with pH. Conversely, the relative abundance of *Firmicutes*, *Proteobacteria*, *Bacteroidetes*, *Fusobacteria* and *Epsilonbacteraeota* showed a decreasing trend with increasing of soil parameters except pH. The Monte Carlo test showed that AK, urease and catalase activity all significantly affected soil bacterial diversity and relative abundance at the phylum level (Figure 5).

## 4 Discussion

Our study found that planting *L. japonica* significantly increased SOC, AN, AP, AK and enzyme activity (Table 1). The improved soil quality parameters might be related to improved soil conditions resulting from greater input of plant residues, especially roots (Abbott et al., 2018; Upadhyay et al., 2022). The accumulation of these nutrients activated soil urease, phosphatase and catalase, boosting microbial activity, metabolism, and activity after planting *L. japonica* in the gravel-mulched land, with faster cycling and transformation of soil nutrients. Planting *L. japonica* increased the plant biomass and subsequent accumulation of the SOC, which could have affected pH (Table 1). It is well-known that soils rich in SOC have a better buffer capacity to adjust the soil pH (Ma et al., 2022). Moreover, the soil quality can be improved relatively quickly, alleviating the problems related to aeolian sandy soil and crop production in the region (Scheibe et al., 2015). There were little to no statistically significant differences in any of those measured soil properties between plots planted 1, 2 or 4 years ago.

Soil properties are among the most important factors shaping microbial communities (Xi et al., 2022). Rhizosphere microbial populations are constantly exposed to both low and high molecular weight organic compounds exuded from roots, which may be affected by specific physical, chemical, and biological conditions and microbial activities (Xie et al., 2019). Current evidence suggests that soil physical and chemical variables have a substantial impact on the microbial abundance and community diversity during land degradation (Li J. et al., 2023). Research on red soil in southern China showed that pH, AP and AN significantly affected soil bacterial community structure (Muneer et al., 2022). Moreover, in the alpine plateau of northern Tibet, variations in soil pH and SOC significantly affected composition of arbuscular mycorrhizal fungal communities (Chen et al., 2022; Zhou et al., 2022; Zhang M. et al., 2022). These studies overlap in their assertion that the change in soil microbial diversity composition and structure may be caused by change in soil characteristics. In the present study, soil nutrients and enzyme activities accounted for 93.63% of the changes in soil bacterial communities, among which available nitrogen, potassium, urease and catalase were significantly affected.

In this study, the most dominant bacterial phyla observed were *Firmicutes*, *Proteobacteria* and *Bacteroidetes* (Figure 3). There is a growing consensus that *Bacteroidetes*, as R-strategy flora, occupy a dominant position in the early stage of microbial community succession, and with the gradual maturity of microbial community succession, the relative abundance of K-strategy flora such as *Acidobacteria* gradually increases (Chen et al., 2016). *L. japonica* treatment resulted in a significant improvement in the relative abundance of *Acidobacteria,* and resulted in a reduction in *Bacteroidetes* and *Fusobacteria* (Figure 3). In this respect, our study revealed that after *L. japonica* planting, the bacterial community transitioned from a rapidly growing copiotrophic group to a slowly growing oligotrophic group. After the cultivation of *L. japonica* in the gravel-mulched field, more plant residues are produced, and a large amount of cellulose is decomposed, creating a favorable environment for the *Acidobacteria* that can use cellulose. *Acidobacteria* have been shown to be closely related to soil pH regulation and the degradation of plant residue polymers (Xu et al., 2020). This finding also accounts for the decrease in pH value to a certain extent (Xi et al., 2022). In addition, *L. japonica* planting in the previous year significantly decreased the relative abundance *of Firmicute*. This finding indicates that *L. japonica* planting for a short-term would reduce the pathogenic bacteria belonging to *Firmicute* (Ehrich et al., 1995).

After *L. japonica* planting, some bacteria at the genus level with relative abundance greater than 1.0% decreased, and those with less than 1.0% abundance increased significantly, which may be related to the significant improvement of soil physicochemical properties and increased bacterial abundance (Urbanová et al., 2015). Growing crops could provide more C and N to the soil for bacterial utilization through residual roots and litter, increase the number of microorganisms, change the bacterial community structure, and promote a change in the soil bacterial community composition.

Soil microbial diversity is a commonly used index to describe the stability of the microbial community, and is informative to fully reflect the significance of the soil environment on the microbial community and assess the health of terrestrial ecosystem (Coyte et al., 2015). Our study showed that *L. japonica* planting increased the number of bacterial OTU (Figure 2) and diversity index (Table 4) in the rhizosphere soil. This occurrence might be ascribed to the fact that rhizosphere soil bacteria can use the nutrients secreted by roots to grow and reproduce in the short term, and the diversity and richness of abundant communities gradually increase after long-term continuous cropping (Zhang H. et al., 2022).

The effect observed in the soils might be mediated primarily via a change in the composition and structure of the vegetation present on both planted and non-planted plots (due to *L. japonica* becoming dominant) (in ‘t Zandt et al., 2023). After planting *L. japonica*, the nutrient substrates such as C, N and P in the soil were affected by roots and litter, and increased significantly (Table 1), promoting bacterial growth in symbiotic groups. As a result of the impact of changes in land use on soil structure and nutrient availability, the configuration of the soil microbial community undergoes alterations (Gschwend et al., 2021; Xi et al., 2022; Labouyrie et al., 2023). A study by Jiang et al. (2022) on the rhizosphere soil of *Torreya grandis* cv. *Merrillii* showed that the effects of pH, organic matter, water-soluble SOC and AN and AP were significant on the dominant soil bacteria.

Overall, *L. japonica* planting significantly changed the soil microbial structure and diversity of the gravel-mulched land by affecting the soil characteristics and contributing significantly in shaping the diversity of microbial communities.

## 5 Conclusion

We found that growing *L. japonica* decreased soil pH, and increased SOC, AN and other nutrients and enzyme activities, with the highest levels observed after 4 years. The improved soil environments gave rise to an increased soil bacterial community diversity. The main dominant phyla of soil bacteria were *Firmicutes*, *Proteobacteria* and *Bacteroidetes*. The relative abundance of *Acidobacteria* increased after planting *L. japonica*, and *Firmicutes*, *Bacteroidetes* and *Fusobacteria* decreased. Collectively, the planting *L. japonica* in the short term could be a low-cost strategy to improve soil nutrients availability and soil bacterial community diversity, and effectively restore degraded land while promoting the local economy in the gravel-mulched area.

## Data availability statement

The authors acknowledge that the data presented in this study must be deposited and made publicly available in an acceptable repository, prior to publication. The names of the repository/repositories and accession number(s) can be found at: NCBI – PRJNA1044469.

## Author contributions

XW and BM developed the concept of this study and are main contributors to writing the manuscript, data analysis, and preparing figures. HL and YB performed all experiments. ML, NM, and LG contributed to the manuscript edit and review. All authors contributed to the article and approved the submitted version.
